# Study on Hydrolysis Properties and Mechanism of Poly(3-Methacrylamido Propyl Trimethyl Ammonium Chloride) Solution

**DOI:** 10.3390/polym14142811

**Published:** 2022-07-10

**Authors:** Yongji Wang, Xu Jia, Yuejun Zhang

**Affiliations:** School of Chemistry and Chemical Engineering, Nanjing University of Science and Technology, Nanjing 210094, China; wyongji2010@126.com (Y.W.); jiaxu@njust.edu.cn (X.J.)

**Keywords:** poly(3-methacrylamido propyl trimethyl ammonium chloride), hydrolysis, mechanism

## Abstract

Poly(3-methacrylamido propyl trimethyl ammonium chloride) (PMAPTAC) is a typical cationic water-soluble polyelectrolyte, which has been widely used in petroleum, papermaking, daily cosmetics and other fields in the form of an aqueous solution. However, the acid–base and thermal stability of PMAPTAC in aqueous solution have not been reported yet, which hinders its further application in high-temperature and acid–base environments. To address these deficiencies, the effects of temperature and pH of PMAPTAC with different intrinsic viscosities on its hydrolysis stability were investigated qualitatively and quantitatively, and the hydrolysis mechanism was studied. Firstly, the qualitative analysis showed that the apparent viscosity of the PMAPTAC solution decreased with hydrolysis time at different temperatures and pH. The higher the temperature and the lower the pH, the greater the viscosity loss of PMAPTAC. The quantitative analysis showed that the hydrolysis rate of the PMAPTAC sample solution increased with the increase in temperature and pH. In addition, the intrinsic viscosity of PMAPTAC samples had little effect on the hydrolytic stability of PMAPTAC. Secondly, by analyzing the viscosity curves at different pH and temperatures by Arrhenius analysis, the Arrhenius equations were found to be 1/τ = 200.34e^((−25.04)/RT), 1/τ = 9127.07e^((−38.90)/RT) and 1/τ = 4683.03e^((−39.89)/RT) for pH 3, pH 7 and pH 11, respectively. Thirdly, the hydrolysis rate of PDMC was the fastest under alkaline conditions. In addition, compared with PDMC, PMAPTAC had better hydrolysis stability under the same conditions. Finally, the mechanism of the hydrolyzed polymer was studied by FTIR and ^13^CNMR, which showed that the carbonyl group of PMAPTAC was hydrolyzed into a carboxyl group, and the small molecule (3-aminopropyl) trimethylammonium chloride was generated, while the ester group of PDMC was hydrolyzed into a carboxyl group, and choline chloride was released. The above results can provide a theoretical basis for the application of PMAPTAC in some high-temperature and acid–base environments.

## 1. Introduction

Poly(3-methacrylamido propyl trimethyl ammonium chloride) (PMAPTAC) is a typical cationic water-soluble polymer polyelectrolyte, which has been widely used in petroleum, textile printing and dyeing, papermaking, daily cosmetics and other fields. In these fields, PMAPTAC is often applied in the form of an aqueous solution, and its application performance is mostly related to the solution properties. Among them, the hydrolytic stability of PMAPTAC is one of the most important solution properties directly related to its various uses and functions.

There have been many reports on the hydrolytic stability of water-soluble polymers, especially the hydrolytic properties of polyacrylamide (PAM) [1,2,3,4,5]. For example, in 1988, Kheradmand et al. [6] studied the hydrolysis of acrylamide–acrylic acid copolymers via potentiometric titration as a function of pH and initial carboxylate content at high temperatures (75 °C~95 °C). The results showed that when pH < 8, the pH and the hydrolysis rate gradually increased with the progress of hydrolysis; when pH > 8, pH and the hydrolysis rate gradually decreased with the progress of hydrolysis. The experimental results confirmed the intramolecular catalysis by neighboring undissociated carboxylate groups for pH < 7 and the catalysis of OH− ions with autoretarded kinetics for 7 < pH < 11. In the same year, Moradi-Araghi et al. [7] found that the imide was the dominant hydrolysis product under acidic conditions [8], and the carboxylate was preferred under neutral and alkaline conditions, as determined via ^13^CNMR. 

However, only a few studies in the literature have reported on the hydrolysis of polyelectrolytes [9,10,11,12]. For example, in 1989, Aksberg et al. [10] investigated the alkaline hydrolysis of a copolymer of acrylamide and acryloyloxyethyltrimethyl ammonium chloride P(AM-DAC) via polyelectrolyte titration and nitrogen analysis in buffered aqueous solutions at different pH ranges (3.5–8.5) and temperatures (22–50 °C). The results of ^13^CNMR analysis showed that choline chloride was released as the hydrolysis product. In addition, the rate of hydrolysis increased with the increase in pH and temperature. This work studied the hydrolytic stability of ester groups and provided a research method that can be used in this study.

In 2005, Fernandez I J et al. [11] investigated the hydrolysis resistance of PMAPTAC and PAM by determining the intrinsic viscosity using a Contraves (LS-30) low shear rotational rheometer at 25 °C. Polymer solutions (1%) were aged in an oven at 120 °C for 30 days after being prepared in NaCl (1M) and bubbled with nitrogen for 10 minutes. The results showed that a slight increase in the pH of PMAPTAC occurred with aging time, and PAM lost 90% of its initial intrinsic viscosity, while PMAPTAC lost only 20% of its initial intrinsic viscosity. Furthermore, the infrared spectra of polymers before and after aging did not show any particular changes that could indicate an important change in structure. This was an earlier report on the hydrolysis of PMAPTAC, but it only investigated the change in the intrinsic viscosity of the PMAPTAC solution with only one molecular weight during the aging time. It failed to investigate not only the effect of temperature, pH and molecular weight on the hydrolysis performance but also its hydrolysis mechanism.

In 2019, Wu J Y et al. [12] studied the hydrolysis resistance of PMAPTAC hydrogel and poly[2-(methacryloyloxy)ethyl trimethyl ammonium chloride] (PDMC) hydrogel at 50 °C under acidic conditions by potentiometric titration. The results showed that the carboxyl group concentration of PDMC hydrogel reached 64.7 mmol/g but only 0.2 mmol/g for PMAPTAC hydrogel after 65 h, implying that the hydrolysis resistance of PMAPTAC was superior to that of PDMC. This paper was the earliest report on the comparative study of the hydrolytic stability of PMAPTAC and PDMC, but it only studied the hydrolysis performance of PMAPTAC and PDMC hydrogels with a single molecular weight at the same pH and temperature and failed to investigate the effects of temperature, pH and molecular weight on their hydrolysis performance, let alone the hydrolysis mechanism of PMAPTAC and PDMC.

From the above literature, it can be seen that there have been only two literature reports on the hydrolytic stability of PMAPTAC samples with a single molecular weight, and the effects of temperature, molecular weight and pH on the hydrolysis performance of PMAPTAC have not been investigated yet, let alone its hydrolysis mechanism, which has led to a lack of comprehensive and profound understanding of the hydrolytic stability of PMAPTAC. Therefore, there is a very urgent need to study the hydrolysis properties and mechanism of PMAPTAC.

In this work, firstly, the hydrolysis performance of PMAPTAC was qualitatively evaluated by measuring the apparent viscosity of sample solutions with different pH at different temperatures using a Brookfield DV2T rotational viscometer. Secondly, the hydrolysis rate of PMAPTAC was quantitatively measured by determining the total nitrogen of the hydrolyzate. Simultaneously, the hydrolytic stability of PDMC was compared with that of PMAPTAC. Finally, a preliminary description of the mechanism of polymer hydrolysis was obtained through the structural characterization of the polymer and the small molecules after hydrolysis by infrared and ^13^CNMR analysis, which lays the foundation for the in-depth study of the structure, properties and applications of PMAPTAC.

## 2. Materials and Methods

### 2.1. Materials and Reagents

**Figure 1:**
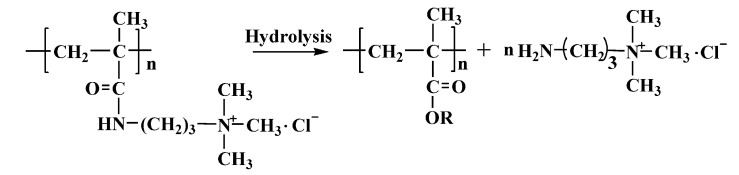
Schematic diagram of possible hydrolysis of PMAPTAC.

**Figure 2:**
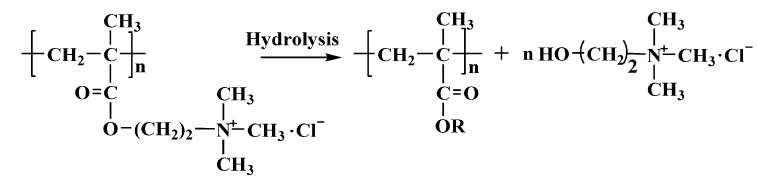
Schematic diagram of possible hydrolysis of PDMC.

Three PMAPTAC colloid samples with serial intrinsic viscosities ([*η*] represents the molecular weight [13], and the unit of [*η*] is dL/g) were synthesized in our lab [13], and they were purified in a water–acetone solvent system to remove residuals. The [*η*] values (determined at 30 ± 0.1 ℃ by using an Ubbelohde viscometer in 1M NaCl) were 0.62, 3.56 and 7.55 dL/g. PDMC with an [*η*] of 3.56 dL/g was also prepared in our lab. The structures are shown in Figure 1 and Figure 2.

The reagents required for the experiment were as follows: nitrogen-free distilled water, potassium persulfate, hydrochloric acid, potassium dihydrogen phosphate, citric acid, sodium hydroxide and potassium chloride, which were all purchased from Chengdu Cologne Chemical Co. Ltd., Chengdu, China, with 99% purity.

The instruments were as follows: Brookfield DV2T rotational viscometer (AMETEK Inc. Brookfield Engineering Laboratories Inc, Middleboro, MA, USA) and LDZX-50KBS autoclave (Shanghai Shen An Medical Instrument Factory, Shanghai, China). 

### 2.2. Solution Preparation

The buffer solutions and hence the pH levels investigated were:

pH 3: 205.5 mL of 0.2 mol/L potassium dihydrogen phosphate and 794.5 mL of 0.1 mol/L citric acid were transferred into a 1L volumetric flask and diluted to volume.

pH 7: 291 mL of 0.1 mol/L sodium hydroxide and 250 mL of 0.2 mol/L potassium dihydrogen phosphate were transferred into a 1 L volumetric flask and diluted to volume.

pH 11: 439 mL of 0.1 mol/L sodium hydroxide and 500 mL of 0.1 mol/L potassium chloride were transferred into a 1L volumetric flask and diluted to volume.

### 2.3. Qualitative Determination of Hydrolysis

The polymer samples were weighed and dissolved in nitrogen-free water to bring their mass concentration to 10 g/L. Then, this solution was added to the above buffer solutions to dilute the mass concentration of the polymer to 1 g/L. After complete dissolution of the polymers in the buffer solutions, the solutions were transferred to glass vials, which were hermetically sealed with tape to prevent water vapor from escaping during heating.

The vials containing the polymer solutions were subjected to aging temperatures of 30 °C, 55 °C and 80 °C. The solutions were removed from the heating system at previously established time intervals, and then the viscosity of the solutions was immediately measured in the viscometer. The solutions were then returned to their respective vials, which were resealed and reheated. 

The apparent viscosity (*η_a_*) of polymer solutions was measured by a Brookfield DV2T rotational viscometer with a 1# rotor at 200 rpm at different pH and temperatures. To better assess changes in the *η_a_* of the polymer solutions over time, the viscosity was expressed as the viscosity retention rate of the solution (*η*%) [14], according to Equation (1). This was defined as the ratio between the viscosity percentage of the polymer solution at any aging time *t* (*η_t_*) and the initial *η_a_* of the same solution (*η_t_* = 0).

(1) $$\eta^{\%}=100\times \frac{\eta_{t}}{\eta_{0}}$$

### 2.4. Arrhenius Analysis

According to the viscosity profiles of the polymer solutions from Section 2.3, viscosity decay constants were obtained for the polymers in solution by performing specific viscosity regression as a function of time [15], as shown in Equation (2).

(2) $$\frac{-t}{\tau }=\ln\left(\frac{\mu_{t}-\mu_{sol}}{\mu_{0}-\mu_{sol}}\right)$$

where *μ_t_* is the specific viscosity at time *t*, *μ*_0_ is the initial specific viscosity, *μ_sol_* is the viscosity of the solvent, and *τ* is the viscosity decay constant (day).

Based on the viscosity decay constants of the polymers for pH 3, pH 7 and pH 11 solutions, Arrhenius analysis was applied according to Equation (3), and the activation energy involved in the polymer degradation process in solution was determined [15].

(3) $$\frac{1}{\tau }=Ae^{\frac{-E_{a}}{RT}}$$

where *R* is the gas constant (8.314 J·mol^−1^·K^−1^), *Ea* is the activation energy (kJ.mol^−1^), *A* is the frequency factor, and *T* is the temperature (K).

According to the viscosity decay constant *τ* and Equation (4), the apparent viscosity and stability of PMAPTAC polymer solutions as a function of temperature and time can be predicted [15].

(4) $$\frac{\mu }{\mu_{0}}=e^{\frac{-t}{\tau }}$$

### 2.5. Principle of Method for Determination of Hydrolysis Rate

According to the hydrolysis mechanism of PAM [16,17,18,19,20] and the hydrolysis mechanism of the ester group [21,22], it was speculated that the structures of PMAPTAC and PDMC hydrolysis products are those shown in Figure 1 and Figure 2, where R is a H or metal ion.

Therefore, after hydrolysis, the polymer solution was dialyzed through a dialysis bag for a period of time. The nitrogen content generated by the hydrolysis of the polymer was determined according to the determination of total nitrogen in water by alkaline potassium persulfate digestion UV spectrophotometry [23], and then the number of moles of nitrogen generated by hydrolysis was calculated and divided by the number of moles of total nitrogen in the sample before hydrolysis. Then, the hydrolysis rate was obtained according to Equation (5):

(5) $$Hydrolysis\;rate^{\%}=100\times \frac{n_{Nt}}{n_{N0}}$$

where *n_N__t_* is moles of nitrogen generated by hydrolysis in time *t*, and *n_N_*_0_ is the total moles of nitrogen in the original sample before hydrolysis.

### 2.6. Research Methods of Hydrolysis Mechanism

In order to obtain a preliminary description of the hydrolysis mechanism of PMAPTAC and PDMC, the dialyzed solution and the polymer in the dialysis bag were freeze-dried, and the obtained products were characterized by infrared and ^13^CNMR after dialysis with a dialysis bag against the polymer solution hydrolyzed for a certain period of time.

## 3. Results and Discussion

### 3.1. Plot of Standard Curve for Determination of Hydrolysis Rate 

**Figure 3:**
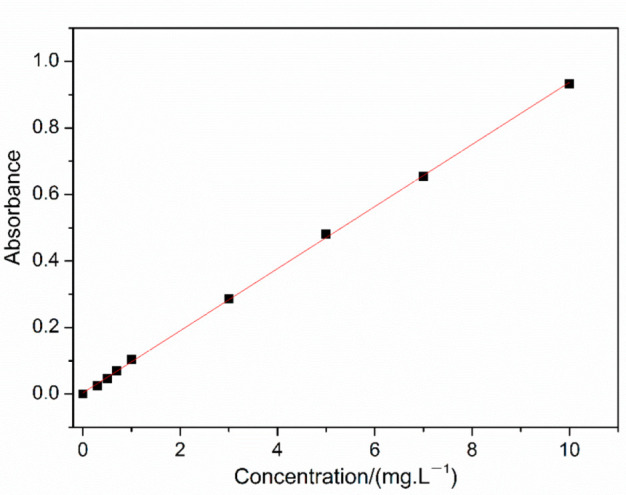
Plot of standard curve.

According to the quantitative determination of hydrolysis in Section 2.5 and the literature [23], 10 mg/L potassium nitrate standard solution was added in volumes of 0.0, 0.1, 0.3, 0.5, 0.7, 1.0, 3, 5, 7 and 10.0 mL to 10 colorimetric tubes and diluted to 10 mL with water. After measuring the absorbance at wavelengths of 220 nm and 275 nm, the standard curve was obtained, as shown in Figure 3. 

It can be seen in Figure 3 that the curve has good linearity in the above concentration range, and the fitted curve equation is y = 0.0934x + 0.035, *R*^2^ = 0.9997, which laid the experimental basis for the quantitative determination of the hydrolysis rate of PMAPTAC and PDMC.

### 3.2. Study on Hydrolysis Properties of PMAPTAC

#### 3.2.1. Effects of Temperature and pH on Hydrolysis Performance of PMAPTAC

(1) Plot of apparent viscosity vs. aging time

**Figure 4:**
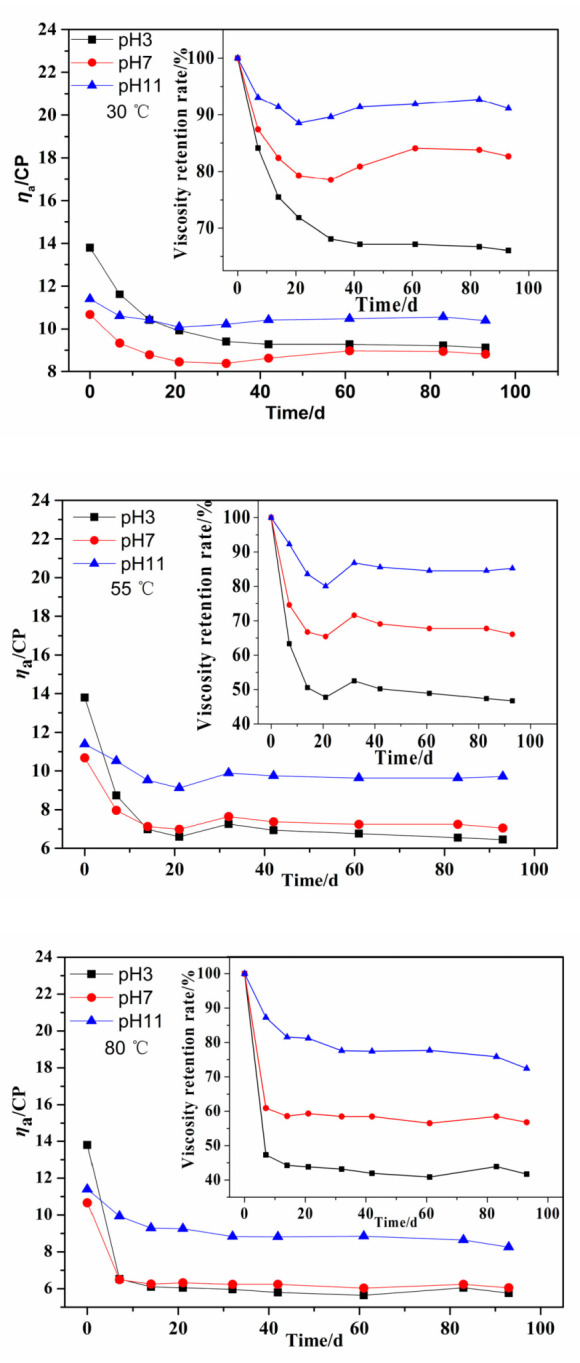
Effect of pH on *η*_a_ of PMAPTAC solution.

PMAPTAC with an [*η*] of 3.56 dL/g was dissolved in buffer solutions at pH 3, 7 and 11. Then, the changes in the apparent viscosity percentage of polymer solutions at 30, 55 and 80 °C and different pH were determined, as observed in Figure 4. 

It can be seen in Figure 4 that the *η*_a_ of the PMAPTAC solution decreased with aging time at different temperatures and pH values. The viscosity retention rates at pH 3, pH 7, and pH 11 at 80 °C after 96 d were 41.74%, 56.79% and 72.43%, respectively, which means that the apparent viscosity loss of the PMAPTAC solution was greater under acidic conditions. When the pH was the same, the higher the hydrolysis temperature, the greater the apparent viscosity loss after 96 d.

(2) Arrhenius analysis

**Figure 5:**
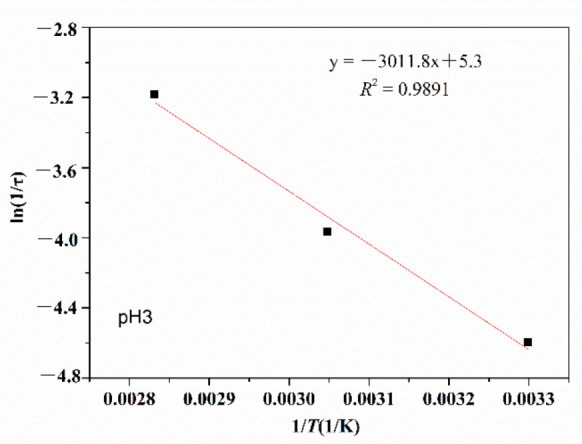
Exponential viscosity decay plots at pH 3.

**Figure 6:**
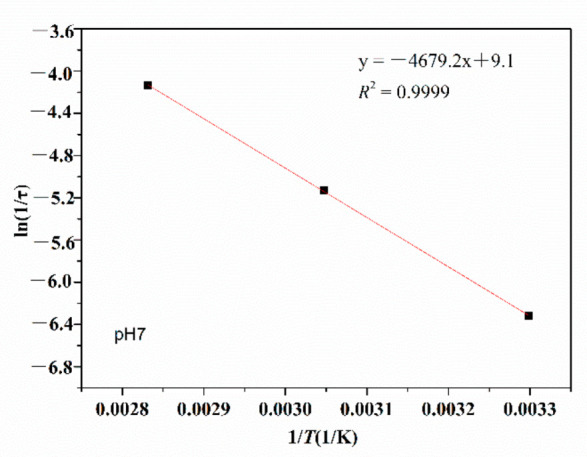
Exponential viscosity decay plots at pH 7.

**Figure 7:**
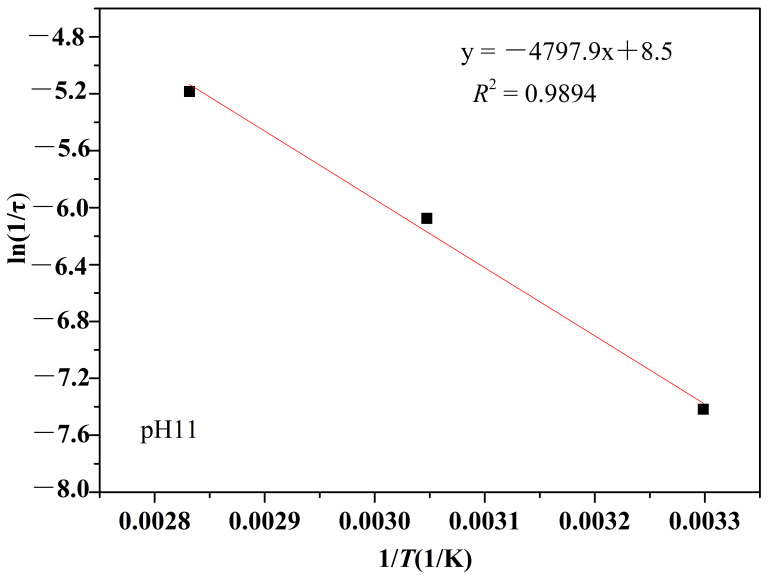
Exponential viscosity decay plots at pH 11.

According to the Arrhenius analysis method in Section 2.4 and the results in Figure 4, exponential decay diagrams of PMAPTAC in pH 3, pH 7 and pH 11 solutions were generated and are depicted in Figure 5, Figure 6 and Figure 7. 

It can be seen in Figure 5, Figure 6 and Figure 7 that the fitted correlation linear coefficients were 0.9891, 0.9999 and 0.9894, respectively. According to the slope of the linear fitting equation in Figure 5, Figure 6 and Figure 7, that is, -*E*a/R, the decay constants and activation energies under pH 3, pH 7 and pH 11 conditions were obtained, and then Equation (3) could be modified to Equations (6)–(8) for pH 3, pH 7 and pH 11, respectively.

(6) $$\frac{1}{\tau }=200.34e^{\frac{-25.04}{RT}}$$

(7) $$\frac{1}{\tau }=9127.07e^{\frac{-38.90}{RT}}$$

(8) $$\frac{1}{\tau }=4683.03e^{\frac{-39.89}{RT}}$$

According to Equations (6)–(8), coupled with Equation (4), the time to reach an apparent viscosity level of 50% of the starting value in pH 3, pH 7 and pH 11 buffer solutions was predicted. For example, for 100 °C, the time to reach an apparent viscosity level of 50% of the starting value was about 11 days, 21 days and 56 days in pH 3, pH 7 and pH 11 buffer solutions, respectively. These results could provide a theoretical basis for the application of PMAPTAC in some high-temperature and acid–base environments.

(3) Quantitative analysis of PMAPTAC hydrolysis

**Figure 8:**
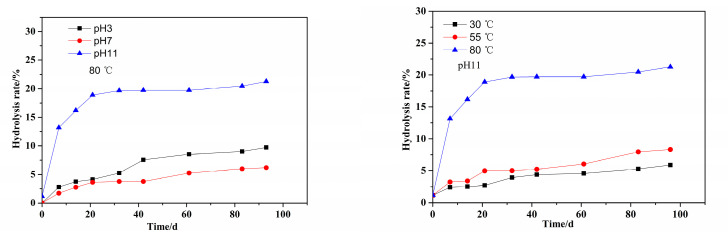
Effects of pH and T on hydrolysis rate of PMAPTAC solution.

According to the determination method of the hydrolysis rate in Section 2.4 and the standard curve in Section 3.1, the hydrolysis rate of the PMAPTAC sample solution was measured at 30, 55 and 80 °C and different pH as a function of time, and the results are shown in Figure 8.

It can be seen in Figure 8 that under the same pH conditions, the hydrolysis rate of the PMAPTAC solution increased with the increase in temperature. For example, for pH 11, the hydrolysis rates at 30 °C, 55 °C and 80 °C after 96 days were 5.88%, 8.32% and 21.27%, respectively. According to the Arrhenius formula [24,25,26], the higher the temperature, the faster the reaction rate and the greater the hydrolysis rate. At the same temperature, the hydrolysis rate of PMAPTAC was higher under alkaline conditions; for example, at 80 °C, the hydrolysis rates at pH 3, pH 7 and pH 11 for 96 d were 9.73%, 6.19% and 21.27%, respectively. It can be seen that the hydrolysis rate under alkaline conditions was higher than that under acidic conditions, which is consistent with the results in the literature [6]. This may be because the PMAPTAC molecular chain is positively charged, and the mutual repulsion of the same charge with H^+^ prevented H^+^ from effectively undergoing nucleophilic addition to the protonated amide carbonyl [27,28,29], resulting in weaker hydrolysis, and finally, the hydrolysis rate of PMAPTAC was lower under acidic conditions. Moreover, it was found that under alkaline conditions, the hydrolysis rate gradually decreased with the progress of hydrolysis, because the OH^−^ ion was the catalyst when the amide was hydrolyzed under alkaline conditions. As the hydrolysis proceeded, the classical repulsion of hydroxide ions by carboxylate anions generated after hydrolysis reduced the concentration of available hydroxide ions near the amide group [6]. Therefore, under alkaline conditions, the catalytic effect of OH^−^ was reduced, resulting in a gradual slowing of the reaction rate as the hydrolysis proceeded; that is, there was a pronounced self-retardation effect.

As can also be seen in the literature [6], the hydrolysis degrees of PAM at 80 °C and pH 3.75, 7.55 and 11.84 were 33%, 12% and 60% after 15 days of hydrolysis, respectively, while the hydrolysis degrees of PMAPTAC after 15 days were 3.74%, 2.77% and 16.19%, respectively, as shown in Figure 8. Thus, it can be concluded that PMAPTAC had good hydrolytic stability compared with PAM. This may be because N-alkyl mono- or di-substitution can significantly improve the hydrolysis resistance of AM chain segments [29,30].

#### 3.2.2. Effect of Molecular Weight of PMAPTAC on Its Hydrolysis Properties

**Figure 9:**
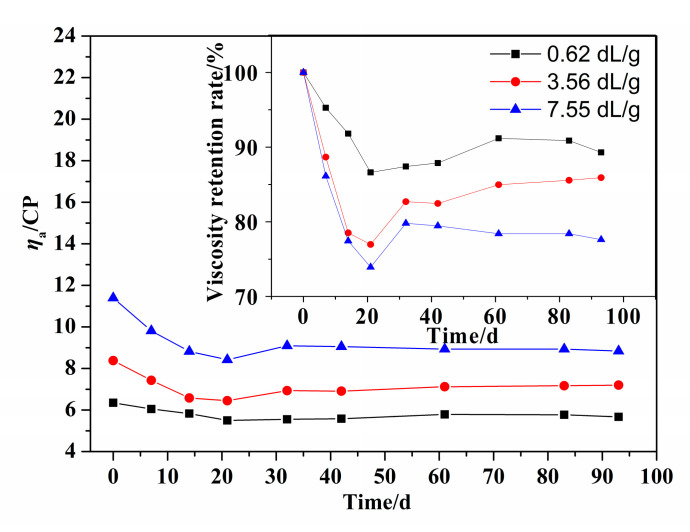
Effect of [*η*] on *η*_a_ of PMAPTAC solution.

**Figure 10:**
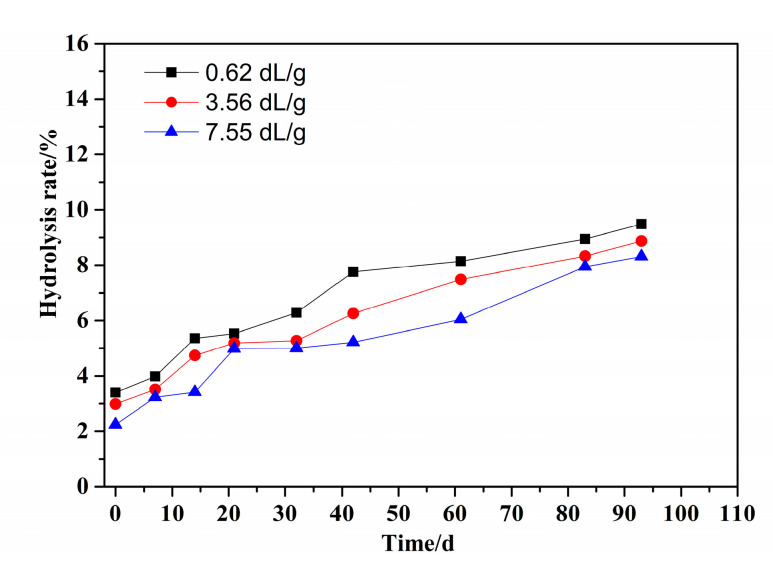
Effect of [*η*] on the hydrolysis rate of PMAPTAC solution.

According to Section 2.3 and Section 2.5, the effect of molecular weight on the hydrolysis properties was investigated when the [*η*] of PMAPTAC was 0.62, 3.56 and 7.55 dL/g. The results are depicted in Figure 9 and Figure 10.

It can be seen in Figure 9 that at the same pH and temperature, the viscosity retention rates of the PMAPTAC solution with an [*η*] of 0.62 and 7.55 dL/g were 92.13% and 78.3% after 96 d, indicating that the degree of hydrolysis of PMAPTAC with 0.62 dL/g was the lowest. This may be related to the degree of curling of the molecular chain in the solution; that is, the larger the [*η*], the longer the molecular chain and the greater the degree of curling in the solution. It can also be seen in Figure 10 that under the same conditions, the hydrolysis rates of PMAPTAC with different [*η*] had little difference after 96d (9.48%, 8.87% and 8.32% for 0.62, 3.56 and 7.55 dL/g), indicating that [*η*] had little effect on the hydrolytic stability.

### 3.3. Comparison with Hydrolysis Performance of PDMC

**Figure 11:**
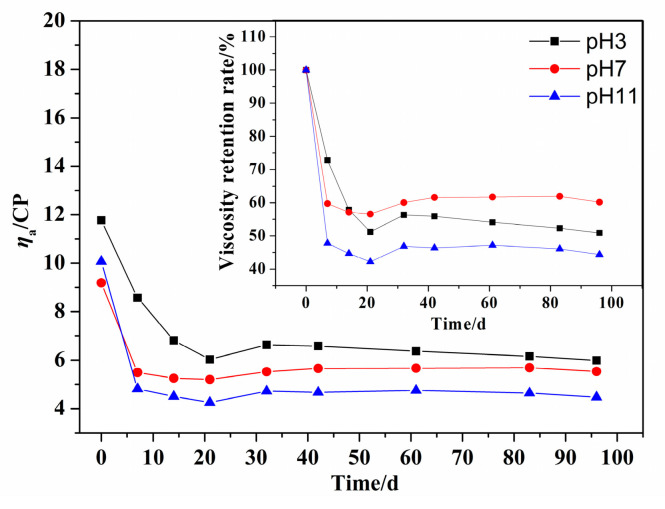
Effect of pH on the *η*_a_ of PDMC solution.

**Figure 12:**
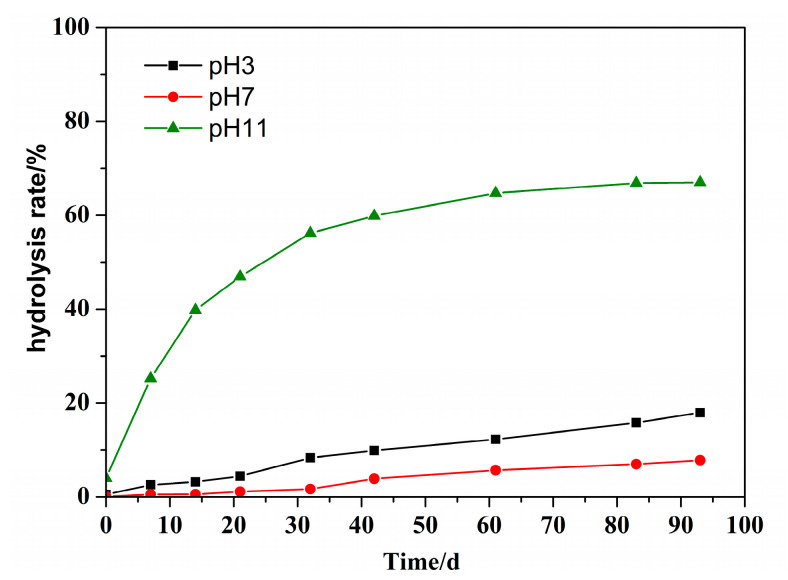
Effect of pH on the hydrolysis rate of PDMC solution.

The molecular structure of PDMC is similar to that of PMAPTAC; the difference between them is that the molecular structure of PDMC contains an ester group. For comparison of their hydrolytic stability, PDMC with the same [*η*] of 3.5 dL/g as PMPATAC was dissolved in buffer solutions (pH 3, 7 and 11). The *η*_a_ and hydrolysis rate of PDMC solutions were determined at 55 °C. The results are shown in Figure 11 and Figure 12, respectively.

It can be seen in Figure 11 that at the same temperature, the viscosity retention rates of PDMC solution under different pH (3, 7, 11) conditions were about 50.89%, 60.17% and 44.40% after 96 d at 55 °C, respectively. Figure 12 shows that the hydrolysis rates were 17.97%, 7.81% and 67.03%, respectively, indicating that the hydrolysis rate of PDMC was the fastest under alkaline conditions. This is because the ester bonds of PDMC molecules are more sensitive to alkaline hydrolysis [2,8].

Furthermore, the curve of the influence of time on the hydrolysis rate was fitted and analyzed; the composite exponential decreasing fitting equation was obtained as y = −62.32exp(−t/17.81) + 66.87, and the correlation coefficient was *R*^2^ = 0.9983. It can be seen that the hydrolysis of PDMC under alkaline conditions gradually decreased with aging time. This may be because the repulsion of hydroxide ions by carboxylate anions generated after hydrolysis reduced the concentration of available hydroxide ions near the ester group. Finally, compared with PMAPTAC, PDMC had a higher hydrolysis rate, indicating that the hydrolysis resistance of PMAPTAC was superior to that of PDMC.

### 3.4. Hydrolysis Mechanism of PMAPTAC

#### 3.4.1. FTIR Spectra 

**Figure 13:**
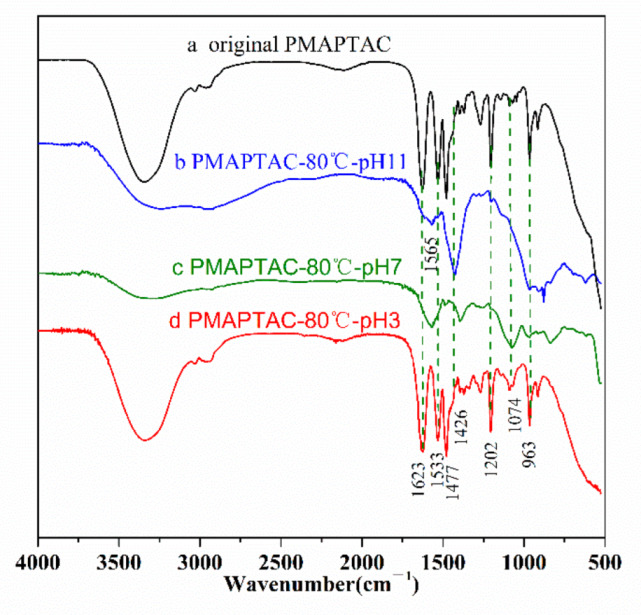
FTIR spectra of the original PMAPTAC and the hydrolyzed product after hydrolysis.

According to the research method of the hydrolysis mechanism in Section 2.6, in order to eliminate the influence of citric acid, the pH 3 buffer solution was replaced with hydrochloric acid (HCl). PMAPTAC solutions hydrolyzed at different pH at 80 °C for 100 days were dialyzed through a dialysis bag, and the hydrolyzed product and the original PMAPTAC sample were characterized by FTIR. The results are shown in Figure 13.

It can be seen in Figure 13 that, first, the C-H stretching vibration absorption peak of methyl and methylene in the polymer chains is found at 2800–3000 cm^−1^. The peaks at 950 cm^−1^ and 1474 cm^−1^ are N^+^(CH_3_)_3_ and methyl stretching vibration peaks on quaternary ammonium, respectively [30], and the absorption peak at 1630 cm^−1^ is assigned to the stretching vibration of C = O in PMAPTAC. The stretching vibration at 1202 cm^−1^ is the C-N stretching vibration of C-N^+^(CH_3_)_3_ [31,32,33]. Secondly, when comparing the spectra of the original PMAPTAC sample and the sample after hydrolysis, the spectrum of the sample after hydrolysis at pH 3 is basically the same as that of the original PMAPTAC sample, and no carboxyl group-related absorption vibration peaks are observed, which may be because the degree of hydrolysis of PMAPTAC under acidic conditions was low, as shown in Figure 8; its degree of hydrolysis was only 9.73%, and possibly, the carboxyl group generated by hydrolysis had a low concentration that was difficult to detect with the infrared spectrometer. Finally, as can be seen in the sample spectra (c, b) after hydrolysis at pH 7 and pH 11, new absorption peaks at 1567 cm^−1^ and 1426 cm^−1^ are observed in the spectra of the samples after hydrolysis, which are attributed to the asymmetric and symmetric stretching vibrations of carboxyl-COO- [34], which indicates that in pH 7 and pH 11 solutions, the amide bond in the molecular structure of PMAPTAC was hydrolyzed into a carboxyl group.

#### 3.4.2. Structural Characterization of Small Molecules Released by PMAPTAC Hydrolysis

**Figure 14:**
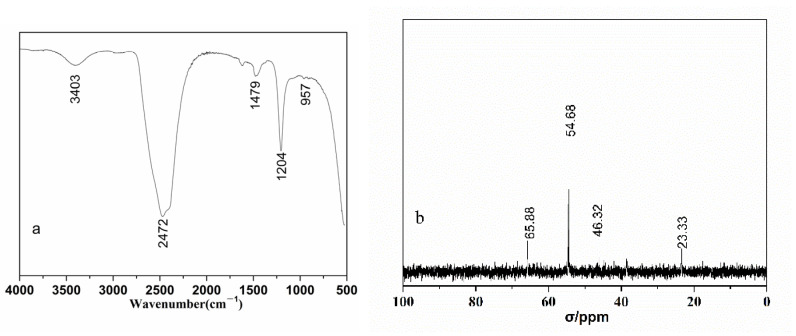
Infrared (**a**) and ^13^CNMR (**b**) spectra of small molecules after PMAPTAC hydrolysis.

The hydrolyzed PMAPTAC solution was treated in a dialysis bag after being heated at pH 3 and 80 ℃, and the dialyzed solution was freeze-dried. Then, the structure was characterized by FTIR and ^13^CNMR. The results are shown in Figure 14.

From the infrared spectrum in Figure 14, it can be seen that the peaks at 957 cm^−1^ and 1479 cm^−1^ are N^+^(CH_3_)_3_ and methyl stretching vibration peaks on quaternary ammonium, respectively [30], and the stretching vibration at 1204 cm^−1^ is the C-N stretching vibration of C-N^+^(CH_3_)_3_ [31,32,33]. The 2472 cm^−^^1^ peak is attributed to the absorption peak of ammonium salt, which may be due to the interaction of HCl and primary amine-NH_2_ on the small molecule generated by the hydrolysis of the amide bond. In addition, it can be seen in the carbon spectrum in Figure 14 that 54.68 ppm is attributed to the peak of the primary carbon atom in the methyl group of N^+^(CH_3_)_3_, and 65.88 ppm, 46.32 ppm and 23.33 ppm are the peaks of the secondary carbon atom in the propyl group. Therefore, the amide bond of PMAPTAC was hydrolyzed into a carboxyl group, and the small molecule (3-aminopropyl)trimethylammonium chloride was released.

#### 3.4.3. Hydrolysis Mechanism Description of PMAPTAC

(1) The acid-catalyzed pathway

**Figure 15:**
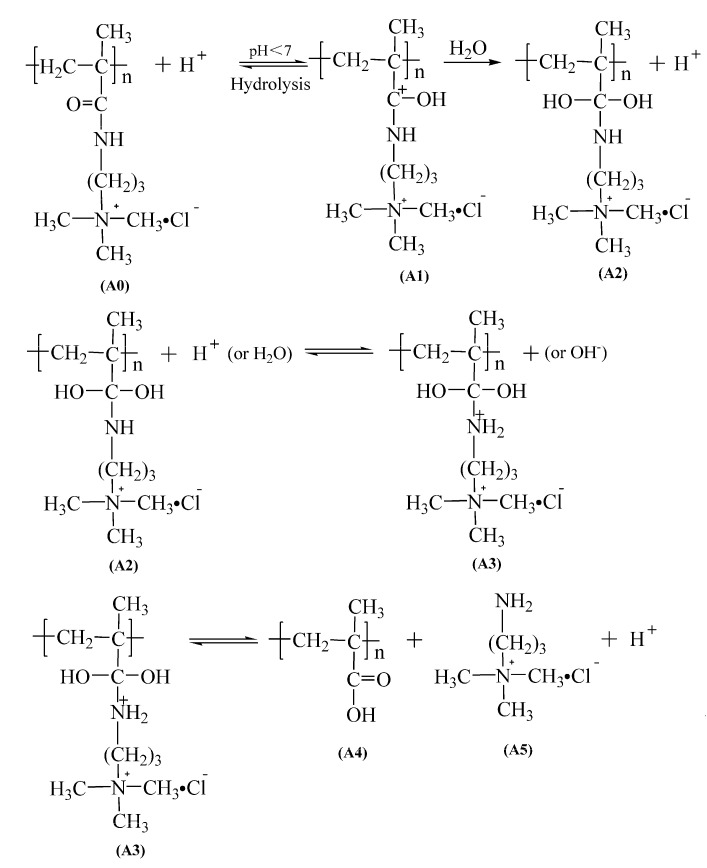
Acid-catalyzed amide hydrolysis mechanism of PMAPTAC.

According to the above results and the literature [16,17,18,19,20], the acid catalysis mechanism of PMAPTAC is shown in Figure 15. The carbonyl carbon atom has a certain positive charge, while the oxygen atom on the carbonyl group has a certain negative charge. Due to the difference in electronegativity between carbon atoms and oxygen atoms, the π and σ bonds on the carbonyl group are distorted, and the π bond also has a certain dipole moment, so the oxygen atom will be partially negatively charged. Eventually, the carbonyl group becomes very vulnerable to attack by hydrogen ions. The detailed hydrolysis process is as follows.

The protonation is commonly believed to occur at the oxygen atom of the carbonyl group after the addition of a proton to the amide (from A0 to A1) [27,35,36]. Then, the protonated amide A1 is the subject of a nucleophilic attack by the oxygen atom of an adjacent water molecule. This water molecule dissociates into OH^−^ and H^+^ when an OH group is added to A1 [28,37]. The excess proton migrates to the solvent in a series of proton-transfer reactions [29,38,39,40]. Then, the nitrogen atom of the intermediate A2 is protonated, which may be carried out either by excess protons from the water phase or by dissociation of a water molecule [28,37]. Finally, the C-N bond of A3 is broken, resulting in the carboxylic acid A4 and the (3-aminopropyl) trimethylammonium chloride A5, accompanied by the deprotonation of the OH group.

(2) The base-catalyzed pathway

**Figure 16:**
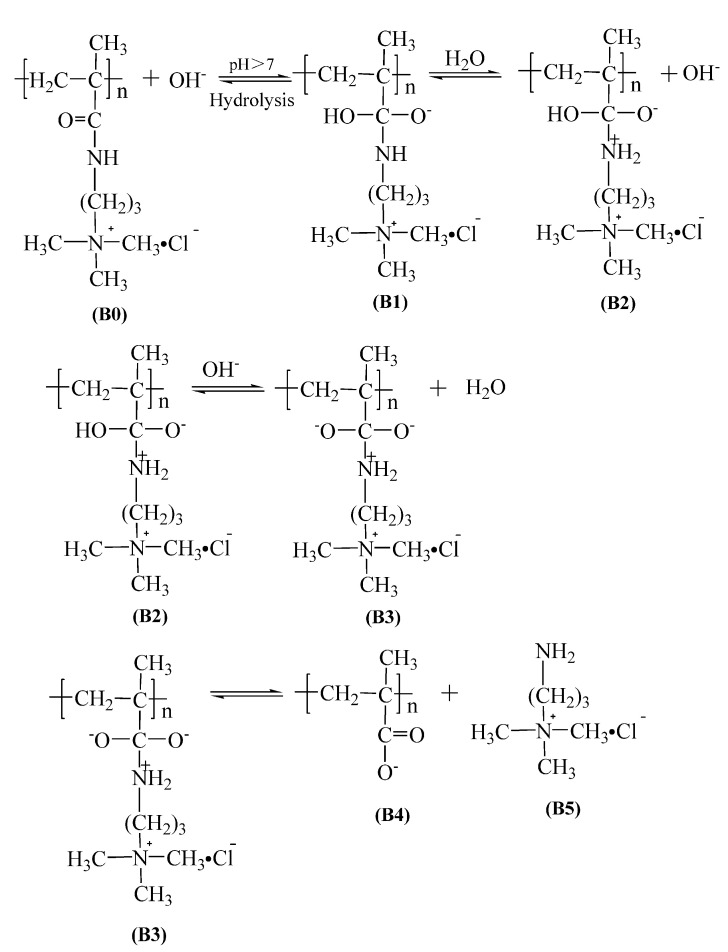
Base-catalyzed amide hydrolysis mechanism of PMAPTAC.

According to the above results and the literature [16,17,18,19,20], the base-catalyzed hydrolysis mechanism of PMAPTAC is shown in Figure 16. Due to the difference in electronegativity between carbon and oxygen atoms, the π and σ bonds on the carbonyl are distorted, and the σ bond also has a certain dipole moment, which will give a partial positive charge to the carbonyl carbon. The detailed hydrolysis process is as follows.

Base-catalyzed amide hydrolysis is initiated by the nucleophilic attack of an OH^−^ ion on the carbon atom of the amide group [41] (from B0 to B1). The intermediate B1 is negatively charged and exhibits a basic character under base conditions, which may induce the dissociation of a solvent water molecule; then, the intermediate B2 is generated through protonation of the nitrogen atom, releasing the solvated OH^−^ ion. In principle, breaking the C-N bond in B2 is possible, but the strongly favored reaction is the deprotonation of the OH group of B2 by an OH^−^ ion, generating the intermediate B3, the C-N bond of which is broken at much lower energy [41]. Finally, the carboxylic acid B4 and the (3-aminopropyl) trimethylammonium chloride B5 are generated by the dissociation of B3.

### 3.5. Hydrolysis Mechanism of PDMC

#### 3.5.1. Structural Characterization of PDMC Polymers 

**Figure 17:**
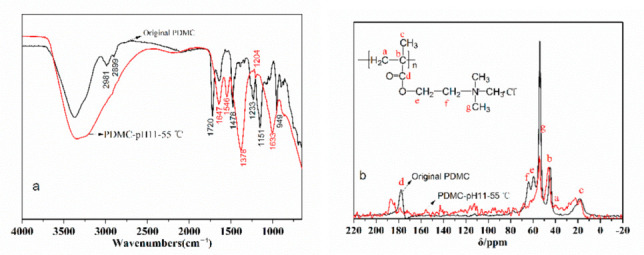
Infrared (**a**) and ^13^CNMR (**b**) spectra of original and hydrolyzed PDMC polymer.

According to the research method of the hydrolysis mechanism in Section 2.6, PDMC hydrolyzed for 100 days at pH 11 at 55 °C was dialyzed through a dialysis bag and then characterized by ^13^CNMR and FTIR. The results are shown in Figure 17.

It can be seen in Figure 17 that, firstly, the peak intensity (53.96 ppm) of the carbon atom in the methyl group of N^+^(CH_3_)_3_ significantly weakened, and the peak intensities (59.48 ppm and 64.15 ppm) of the carbon atoms in the methylene group in the side chain also weakened or disappeared. Secondly, the peak intensities (18.41 ppm, 35.70 ppm and 44.80 ppm) of the associated secondary, tertiary and quaternary carbon atoms in the main chain were basically unchanged [34], and finally, the peak intensities of the carbon atoms in the carbonyl group in the ester group (177.75 ppm) became weaker. The new peak at 186.68 ppm is attributed to the carbon atom in the carboxyl group [34].

It can also be seen in Figure 17 that the peaks at 950 and 1474 cm^−1^ are N^+^(CH_3_)_3_ and methyl stretching vibration peaks on quaternary ammonium, respectively [30], and the absorption peak at 1720 cm^−1^ is attributed to the stretching vibration of C=O in PDMC [42,43]. By comparing the spectra of the original PDMC sample and the sample after hydrolysis at 80 °C and pH 11, it can be seen that after hydrolysis, the peaks at 950 cm^−1^ and 1478 cm^−1^ related to quaternary ammonium salts disappeared or weakened, while the peak at 1720 cm^−1^ related to esters also disappeared. In particular, new or significantly enhanced absorption peaks appeared at 1548 cm^−1^ and 1378 cm^−1^, which are attributed to the asymmetry and symmetry of carboxyl-COO- stretching vibration [34]. Therefore, during the alkaline hydrolysis process of PDMC, the ester group in the molecule was hydrolyzed into the carboxyl group.

#### 3.5.2. Structural Characterization of Small Molecules Released from PDMC Hydrolysis

**Figure 18:**
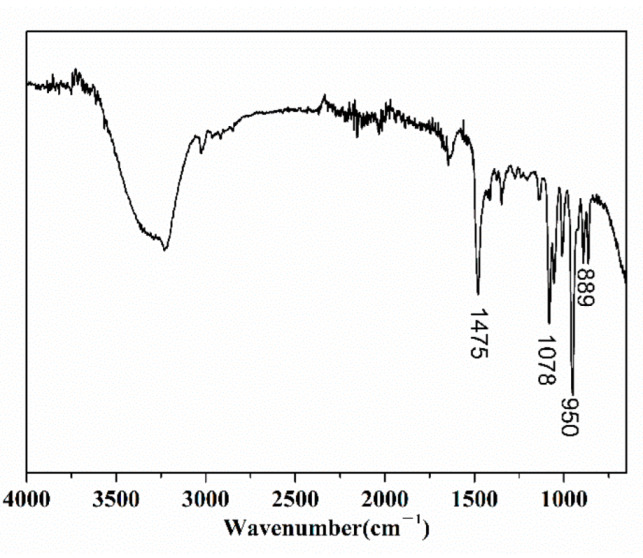
FTIR spectrum of small molecules released by PDMC hydrolysis.

After the hydrolyzed PDMC solution was treated in a dialysis bag, the dialyzed solution was freeze-dried, and then the structure was characterized by FTIR. The result is shown in Figure 18.

**Figure 19:**
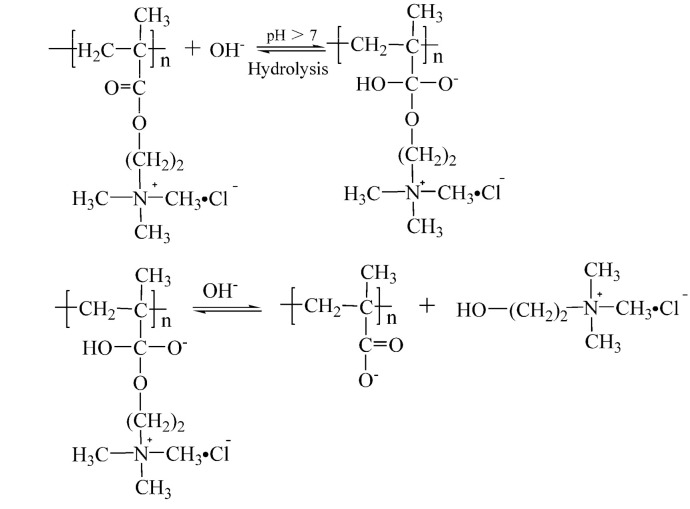
Base-catalyzed ester hydrolysis mechanism of PDMC.

Figure 18 shows that the peaks at 950 cm^−1^ and 1475 cm^−1^ are N^+^(CH_3_)_3_ and methyl stretching vibration peaks on quaternary ammonium, respectively [30], and the stretching vibration at 1078 cm^−1^ is the CH_2_CH_2_-O stretching vibration. It can be seen that the FTIR spectrum of the small molecules after hydrolysis of PDMC are consistent with the spectrum of choline chloride [30], thus proving that choline chloride was generated after PDMC hydrolysis. In summary, during the alkaline hydrolysis process of PDMC, when the ester group in the molecule was hydrolyzed, the small molecule choline chloride was generated, and the ester group of the side chain was hydrolyzed into a carboxyl group. This result is consistent with that reported in the literature [9,20]. The basic hydrolysis mechanism of PDMC is shown in Figure 19. 

## 4. Conclusions

(1) Qualitative analysis showed that the apparent viscosity of PMAPTAC solution decreased with hydrolysis time at different temperatures and pH. The lower the pH and the higher the temperature, the greater the apparent viscosity loss of the PMAPTAC solution. Quantitative analysis showed that the hydrolysis rate of PMAPTAC sample solution increased with the increase in temperature and pH. For example, the hydrolysis rates over 96 d at 30℃, 55 ℃ and 80 ℃ at pH 11 were 5.88%, 8.32% and 22.07%, respectively. In addition, the hydrolysis rates of PMAPTAC samples with different [*η*] were not much different, indicating that [*η*] had little effect on the hydrolytic stability of PMAPTAC.
(2) By analyzing the viscosity curves at different pH and temperatures by Arrhenius analysis, the Arrhenius equations for pH 3, pH 7 and pH 11 were 1/τ = 200.34e^((−25.04)/RT), 1/τ = 9127.07e^((−38.90)/RT) and 1/τ = 4683.03e^((−39.89)/RT), respectively. PMAPTAC solutions were projected to maintain at least half their original viscosity for over 11 days at pH 3, 21 days at pH 7, and 56 days at pH 11, which were predicted according to the formula.
(3) The apparent viscosity of the PDMC polymer decreased with aging time. The viscosity retention rates were 50.89%, 60.17% and 44.40%, and the hydrolysis rates of PDMC solution were 17.97%, 7.81% and 67.03% in pH 3, pH 7 and pH 11 solutions after 96 d at 55 ℃, respectively, indicating that the hydrolysis rate of PDMC was the fastest under alkaline conditions. In contrast, PMAPTAC had significantly superior hydrolytic stability under the same conditions
(4) The mechanism of the hydrolyzed polymer was studied by FTIR and ^13^ CNMR, which showed that the carbonyl group of PMAPTAC in solution was hydrolyzed into a carboxyl group, and the small molecule (3-aminopropyl) trimethylammonium chloride was generated, while the ester group of PDMC was hydrolyzed into the carboxyl group, and choline chloride was released. The above results can provide a theoretical basis for the application of PMAPTAC in some high-temperature and acid–base environments.

## Data Availability

Data presented in this study are available on request from the first author.
